# Churg-Strauss syndrome associated with AA amyloidosis: a case report

**Published:** 2012-06-11

**Authors:** Mouna Maamar, Zoubida Tazi-Mezalek, Hicham Harmouche, Zitouna El Hamany, Mohammed Adnaoui, Mohammed Aouni

**Affiliations:** 1 Department of Internal Medicine Ibn Sina Hospital Rabat Morocco; 2 Department of Nephrology Ibn Sina Hospital Rabat Morocco

**Keywords:** Churg-Strauss syndrome, amyloidosis, vasculitis, purpura

## Abstract

Churg Strauss syndrome is a rare systemic and pulmonary vasculitis exceptionally associated with AA amyloidosis. We report the case of a 65-year old woman with past medical history of asthma. She developed polyarthralgia, headache and purpura. A laboratory workout found hypereosinophilia (1150/µL), positive p-ANCA, microscopic haematuria and proteinuria at 2g/day. A diagnosis of Churg-Strauss syndrome was established based on five criteria of the American College of Rheumatology (ACR). Renal biopsy showed an important type AA amyloid deposit. The patient was treated with steroids with a good response of the vasculitis and amyloidosis with disappearance of the proteinuria.

## Introduction

Churg Strauss syndrome is a rare systemic and pulmonary vasculitis defined by the association of severe asthma, hypereosinophilia of the blood and tissues and vasculitis [1]. We report an exceptional case of Churg-Strauss syndrome associated with renal amyloidosis AA.

## Patient and observation

A 65-year old Moroccan woman was hospitalized for polyarthralgia and frontal headache. She had a past medical history of asthma for 10 years and was operated for hydatid cyst of the liver in 1998. She has no history of tuberculosis, no chronic abdominal pain.

On physical examination, blood pressure was 140/70 mmHg and the heart rate 70/min. She has no fever and the peripheral pulses were all present. We noted an infiltrated purpura in the lower extremities and a wheezing in the pulmonary fields. She has areflexia and sensory involvement in the lower limbs.

Hemoglobin was at 7.5 g/dl, MCV 80 fl, Reticulocyte count at 47.10X3/mm3, platelet count at 730 000/ µL (Normal 150–450 000) and white blood cell count 10000/ µL with neutrophil 4300, lymphocytes at 3600/ µL and eosinophil 1150/ µL (normal 100–500/ µL). Erythrocyt sedimentation rate was 130 mm first hour, C-reactive protein 38 mg/l (range < 6 mg/l), fibrinogen 6 g/l (2–4 g/l) and serum protein electrophoresis showed a polyclonal IgG 24 g/l (range 9–13 g/l) with normal immunofixation. There was renal failure with urea 3 mmol/l (normal 3 – 8.5), creatinemia 160 µmol/l (normal 45 -100) and creatinine clearance at 30 ml/min (Cockcroft formula).

Urinalysis showed a microscopic haematuria at 20 000 cell/mm3 and proteinuria at 2.5g/24Hr. Cryoglobulinemia was negative, p-ANCA were present. Electromyography showed an axonal polyneuropathy of the 4 limbs. Skin biopsy revealed leukocytoclastic vasculitis with extra-vascular eosinophil. Thorax CT showed pulmonary infiltrates changes. Brain CT, temporal artery biopsy and ocular examination were all normal. Renal biopsy did not reveal vasculitis or inflammatory process, but an important type AA amyloid deposit. Salivary biopsy also showed AA amyloid deposit. She has no Familial Mediterranean fever, no history of tuberculosis or other chronic infections.

The diagnosis of Churg Strauss associated with AA amyloidosis was established with five criteria of the American College of Rheumatology (ACR).

Steroids were administered at a pulse of methyprednisolone 1g/day for 3 days followed by oral prednisone at 1 mg/kg/day for 4 weeks and progressively tapered. The patient was also treated with captopril. The clinical and biological response was obtained with disappearance of pain; eosinophil count decrease to 300/µL and negativity of proteinuria. At 26 months follow-up proteinuria remained negative.

## Discussion

AA amyloidosis is a classical complication of longstanding chronic inflammatory conditions such as rheumatoid arthritis, ankylosing spondylitis, chronic infections such as tuberculosis, osteomyelitis or during periodic fever syndrome such as Familial Mediterranean fever [2].

In the largest prospective series of 374 AA amyloidosis, the most frequent underlying disorder was rheumatoid arthritis (33%) and juvenile idiopathic arthritis (17%) [3]. Vasculitis was reported in only four patients [3]. Among vasculitis, Behcet's disease is the most frequent cause of amyloidosis [4].

In our patient, we excluded the most frequent causes of amyloidosis such as tuberculosis, rheumatoid arthritis and she has no Familial Mediterranean fever. To our knowledge this is the first report of renal amyloidosis associated with the Churg-Strauss syndrome. Only one other case of Churg-Strauss syndrome with localized conjonctival amyloidosis has been reported in the literature as an abstract [5]. This association is exceptional because inflammatory process is rare in Churg-Strauss syndrome. This disease is characterized by a strong Th2 type immune response with production of Interleukin 13, 14 and 15 cytokines [6]. Interleukin-5 is one of the key inducers of eosinophilia [7]. Thus, it is very important to first exclude the most frequent causes of renal amyloidosis.

## Conclusion

Churg-Strauss syndrome is a rare vasculitis which was exceptionally complicated by AA amyloidosis. Renal involvement is uncommon in this vasculitis and should encourage realizing renal biopsy.
